# A longitudinal and transancestral analysis of DNA methylation patterns and disease activity in lupus patients

**DOI:** 10.1172/jci.insight.143654

**Published:** 2020-11-19

**Authors:** Patrick Coit, Lourdes Ortiz-Fernandez, Emily E. Lewis, W. Joseph McCune, Kathleen Maksimowicz-McKinnon, Amr H. Sawalha

**Affiliations:** 1 Division of Rheumatology, Department of Pediatrics, University of Pittsburgh, Pittsburgh, Pennsylvania, USA.; 2 Graduate Program in Immunology and; 3 Division of Rheumatology, Department of Internal Medicine, University of Michigan, Ann Arbor, Michigan, USA.; 4 Division of Rheumatology, Henry Ford Health System, Detroit, Michigan, USA.; 5 Division of Rheumatology and Clinical Immunology, Department of Medicine, University of Pittsburgh, Pennsylvania, USA.; 6 Lupus Center of Excellence, University of Pittsburgh School of Medicine, Pittsburgh, Pennsylvania, USA.; 7 Department of Immunology, University of Pittsburgh, Pittsburgh, Pennsylvania, USA.

**Keywords:** Autoimmunity, Epigenetics, Lupus

## Abstract

Epigenetic dysregulation is implicated in the pathogenesis of lupus. We performed a longitudinal analysis to assess changes in DNA methylation in lupus neutrophils over 4 years of follow-up and across disease activity levels using 229 patient samples. We demonstrate that DNA methylation profiles in lupus are partly determined by ancestry-associated genetic variations and are highly stable over time. DNA methylation levels in 2 CpG sites correlated significantly with changes in lupus disease activity. Progressive demethylation in *SNX18* was observed with increasing disease activity in African American patients. Importantly, demethylation of a CpG site located within *GALNT18* was associated with the development of active lupus nephritis. Differentially methylated genes between African American and European American lupus patients include type I IFN–response genes such as *IRF7* and *IFI44,* and genes related to the NF-κB pathway. *TREML4*, which plays a vital role in TLR signaling, was hypomethylated in African American patients and demonstrated a strong *cis*–methylation quantitative trait loci (*cis-*meQTL) effect among 8855 *cis*-meQTL associations identified in our study.

## Abstract 2

A genome-wide DNA methylation and genotyping approach in neutrophils from lupus patients identifies methylation changes that occur with time and as disease activity changes.

## Graphical abstract

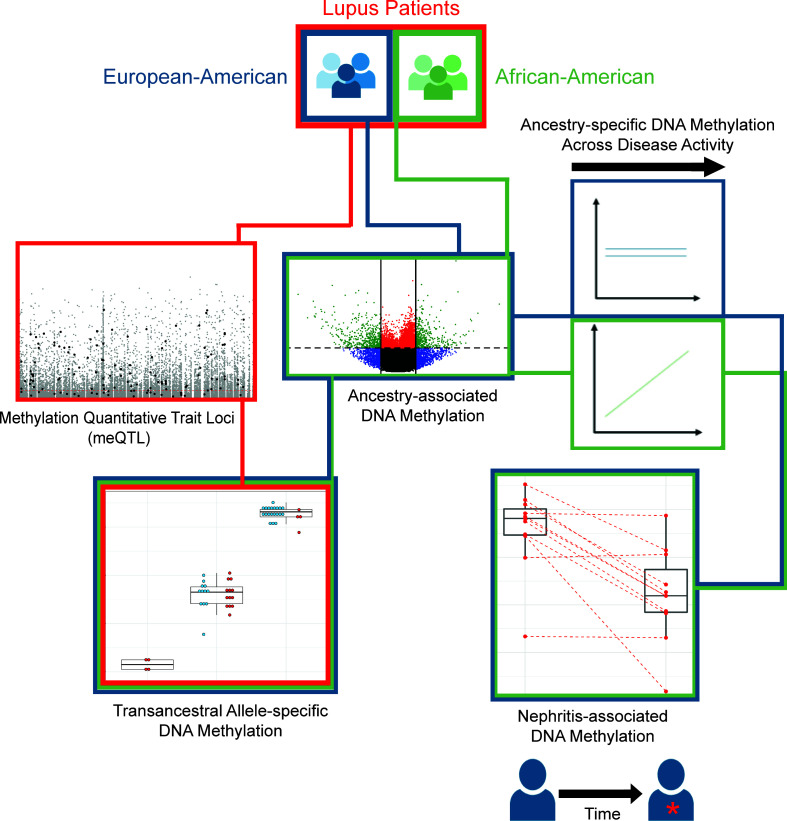

## Introduction

Systemic lupus erythematosus (SLE or lupus) is an autoimmune disease of incompletely understood etiology. Genetic, epigenetic, and environmental factors are thought to play key roles in the immune dysregulation underlying the development of the disease (1). Lupus is characterized by the production of autoantibodies against nuclear antigens and a remitting-relapsing disease course that can target multiple organ systems (2). Frequent disease flares and prolonged periods of active disease are associated with a more deleterious outcome in lupus patients and a higher risk of tissue and organ damage (3).

Lupus is associated with changes in gene expression, including prominent type I IFN and neutrophil gene signatures in the peripheral blood (4–7). Furthermore, increased disease activity in lupus is associated with transcriptional profiles implicating different innate and adaptive peripheral immune cells in individual patients followed longitudinally (7). Notably, progression to active nephritis in lupus patients was associated with gradual enrichment in neutrophil transcripts (7). Indeed, a prominent role for neutrophils in the pathogenesis of lupus is being more clearly elucidated (8).

DNA methylation, an epigenetic mechanism that regulates gene expression, is altered in the immune cells of lupus patients and is potentially influenced by both environmental and genetic factors (9). DNA methylation defects in lupus are suggested to promote an overactive immune response when exposed to inflammatory signals like autoantibody-autoantigen complexes or endogenous nucleic acids (10–12). Methylation quantitative trait loci (meQTL) are genetic polymorphisms that are associated with DNA methylation either directly through alteration of CpG dinucleotides or at a distance through an intermediary process. meQTL identified in prior lupus studies show enrichment for lupus susceptibility genes and type I IFN response genes, suggesting that altering DNA methylation levels at specific loci could be a potential mechanism by which risk alleles contribute to disease susceptibility in lupus (13–15). Lupus susceptibility is significantly higher in patients of non-European ancestry, who are also more likely to develop more severe disease, even after accounting for the influence of social and environmental factors (16). Thus, meQTL analysis provides a potential approach to better understand the mechanisms underlying the observed differences in disease manifestations and outcomes in lupus patients of different ancestries.

Recent work investigating DNA methylation changes in lupus and associated downstream effects and underlying upstream regulatory mechanisms have resulted in significant insights into the pathogenesis of lupus and the identification of novel therapeutic targets for the disease (17). Cell type–specific precision delivery systems to modify the epigenome are promising approaches in the treatment of autoimmune diseases including lupus (18). Furthermore, DNA methylation changes have been suggested as diagnostic markers and markers that can potentially predict specific disease manifestations in lupus (11, 19–21). However, DNA methylation studies in lupus to date have been cross-sectional, and longitudinal studies investigating epigenetic changes in patients with lupus over time have not been reported.

We have previously demonstrated robust demethylation of IFN-regulated genes in lupus neutrophils compared with normal healthy controls (12). In this study, we investigate neutrophil DNA methylation changes over time and across disease activity levels in a cohort of lupus patients followed longitudinally for up to about 4 years. Moreover, we sought to increase our understanding of how DNA methylation is affected by the genetic background. We compared DNA methylation patterns between African American and European American lupus patients, performed meQTL analyses in lupus neutrophils, and identified CpG sites that show methylation changes correlating with disease activity and the development of lupus nephritis across the course of the disease.

## Results

### Longitudinal analysis of DNA methylation over time and across disease activity levels in lupus patients.

**Figure 1:**
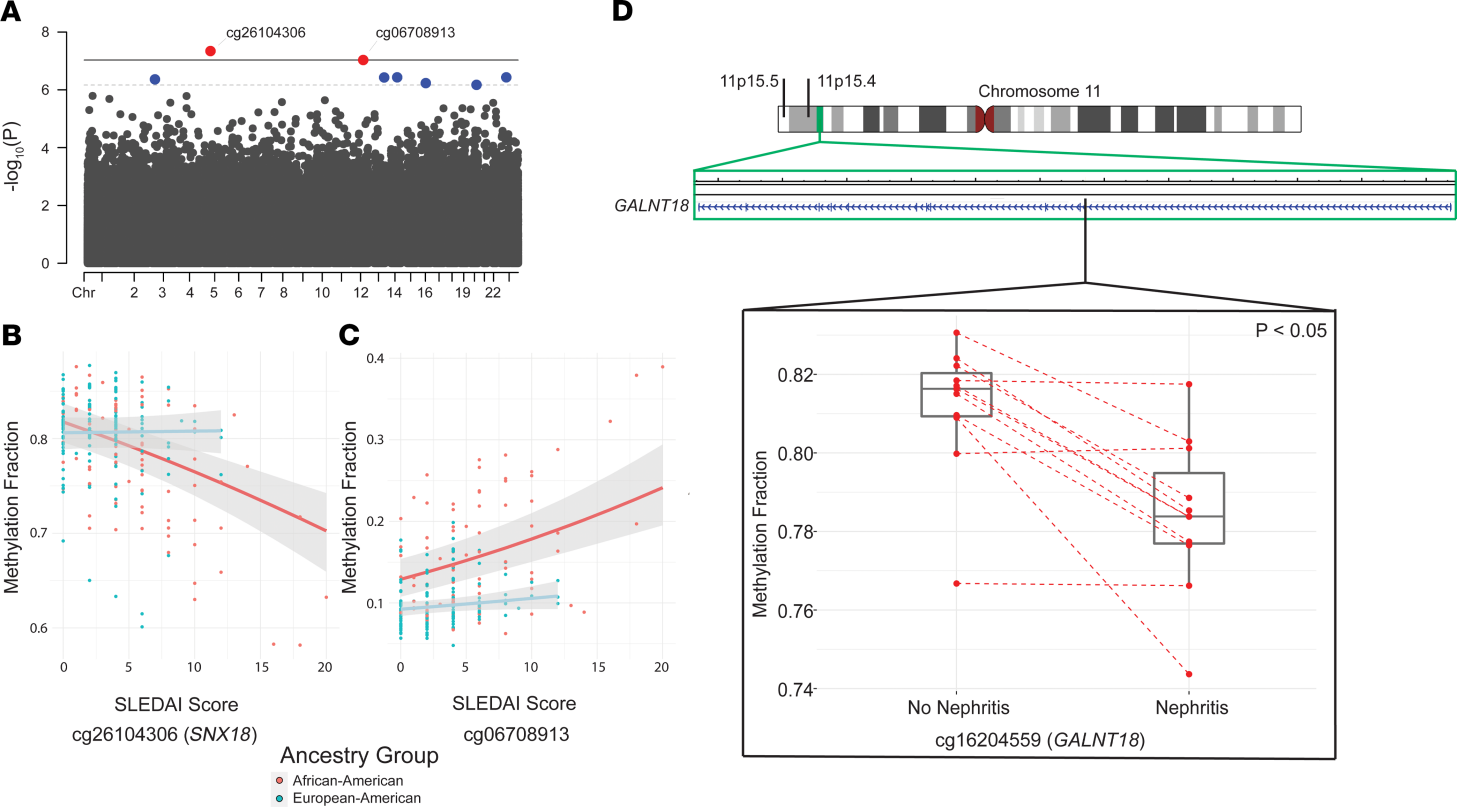
The relationship between DNA methylation changes, disease activity, and the development of lupus nephritis in a longitudinal cohort of lupus patients. (**A**) A Manhattan plot depicting the significance of correlation between methylation levels of CpG sites and disease activity as measured using SLEDAI scores in African American lupus patients (*n* = 93 samples). The red dots are CpG sites that meet the threshold for significance of FDR-adjusted *P* < 0.05 (bold line), and the blue dots are CpG sites that meet the suggestive threshold of FDR-adjusted *P* < 0.10 (dashed line). (**B** and **C**) Methylation status of cg26104306 (**B**) and cg06708913 (**C**) across SLEDAI scores for African American (*n* = 93 samples; red dots/line) and European American (*n* = 136 samples; blue dots/line) lupus patients. (**D**) Ideogram of chromosome 11 showing the location of 11p15.5 and 11p15.4 cytobands. Cg16204559 (black line) is within the body of *GALNT18* located in the 11p15.4 region (green box). Methylation profiles for *n* = 11 lupus patients (red dots; *n* = 7 African American and *n* = 4 European American) at a time point with active nephritis and the nearest preceding or receding time point without nephritis were compared after adjusting for medications, age, and ancestry group using a linear mixed effects model. Cg16204559 (*GALNT18*) was significantly demethylated (FDR-adjusted *P* = 0.048) with the occurrence of nephritis. Mean β nephritis was 78.4% (25th percentile, 80.9%; 75th percentile, 82.0%) and mean β nonnephritis was 81.2% (25th percentile, 77.7%; 75th percentile, 79.5%). Whiskers extend to the maximum value within 1.5 times the IQR on either end of the group. Points beyond the whiskers are outliers.

The Infinium MethylationEPIC array measures the methylation status of 866,836 methylation sites across the genome, including 863,904 CpG and 2932 CNG sites (C, cytosine; N, any nucleotide; G, guanine) (22). After QC and technical probe masking, a total of 745,477 (86.0%) sites were retained for analysis. Heterogeneity in disease manifestations, patient genetic background, and the environment are all factors that complicate the understanding of lupus pathogenesis. Using repeated sampling of lupus patients followed longitudinally, we can account for these factors and detect potentially novel changes in DNA methylation that are associated with disease activity over time. We followed a total of 54 lupus patients for up to 43 months and assessed genome-wide DNA methylation levels in neutrophils in a total of 229 patient samples. Our cohort included 22 African American and 32 European American lupus patients followed across 93 and 136 time points, respectively. We assessed correlation between DNA methylation changes in individual methylation sites across the genome with disease activity as measured by Systemic Lupus Erythematosus Disease Activity Index (SLEDAI) scores in each ancestry group. After removing CpG-SNP probes with a minor allele frequency > 1% to avoid a bias due to intraancestral allele frequency differences, we analyzed a total of 733,192 (84.6%) methylation sites. In the African American cohort, we identified a total of 8 CpG sites that met our suggestive FDR-adjusted *P* < 0.1 (Figure 1A and Table 1). Two sites — cg26104306 (*SNX18*; FDR-adjusted *P* = 3.38 × 10^–2^) and cg06708913 (FDR-adjusted *P* = 3.43 × 10^–2^) — were significantly associated with changing disease activity levels in our cohort (Figure 1, B and C). Cg26104306 shows stark demethylation with increasing disease activity compared with our European American patients, who showed very little methylation change across time and disease activity. Similarly, cg06708913 shows a much higher rate of increasing methylation with disease activity in African American patients relative to European American patients. The inclusion of the top 4 medication components as fixed effects did not improve the fit of our model for these 2 CpG sites (cg26104306 χ^2^ *P* = 0.25 and cg06708913 χ^2^ *P* = 0.83). Our European American sample cohort analysis did not identify any CpG-SLEDAI score associations at either *P* value threshold. Importantly, these data suggest that DNA methylation patterns defining lupus patients are largely stable over time and across disease activity.

We next performed an analysis in a subset of patients who developed active lupus nephritis at any time point during our study and in whom a sample from at least 1 time point without evidence of lupus nephritis is available. After adjusting for medications, age, and ancestry, we identified a single CpG site with a statistically significant relationship between DNA methylation levels and active nephritis in *n* = 11 lupus patients. DNA methylation levels in cg16204559, which is located within the gene *GALNT18*, are significantly reduced during active nephritis in lupus patients (Supplemental Table 1 and Figure 1D; supplemental material available online with this article; https://doi.org/10.1172/jci.insight.143654DS1).

### DNA methylation differences in neutrophils of African American and European American lupus patients.

**Figure 2:**
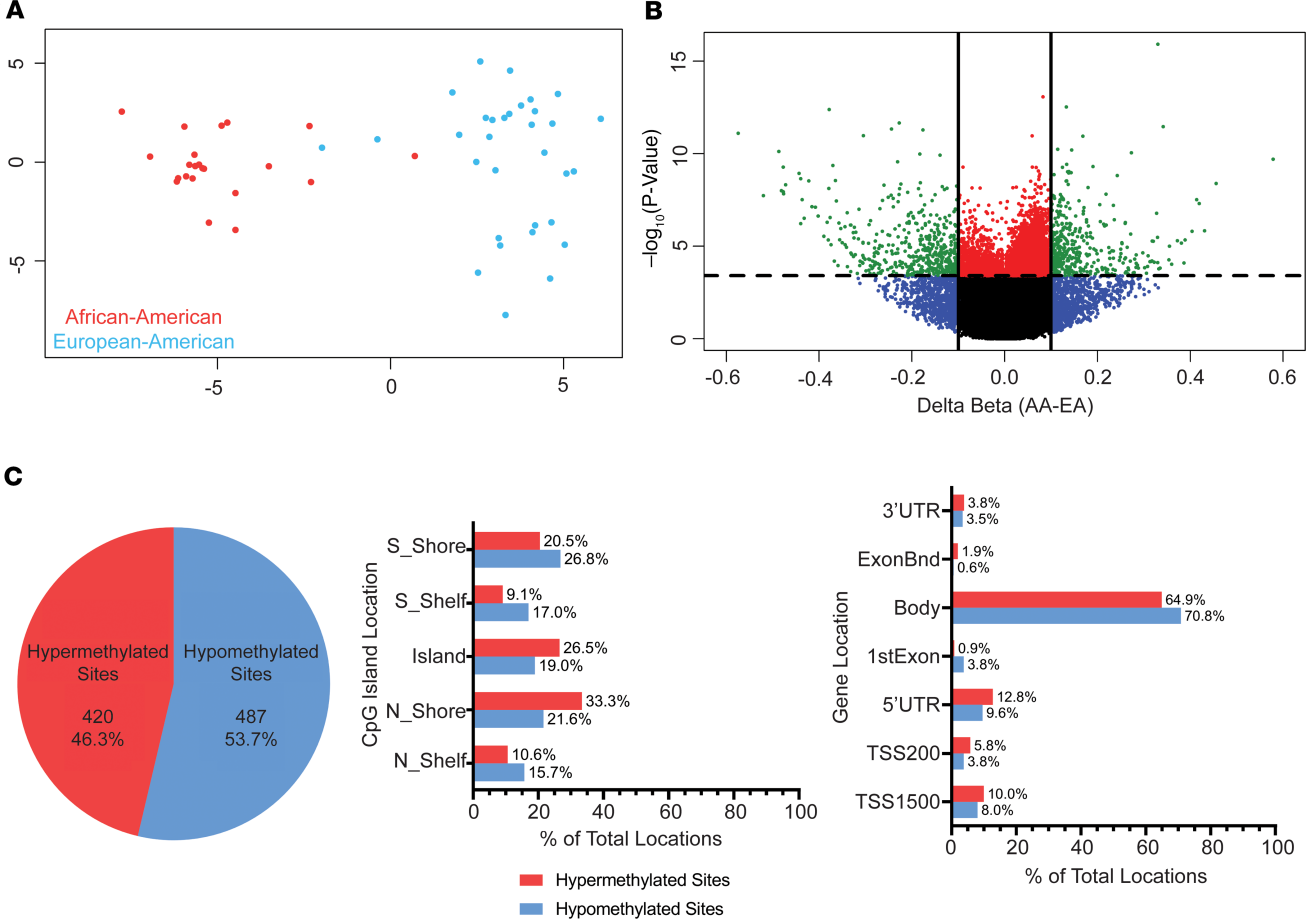
Neutrophils of African American and European American lupus patients show DNA methylation differences associated with ancestry. (**A**) Multidimensional scaling plot of top 5000 most variable CpG sites in African American (*n* = 22; red circles) and European American (*n* = 32; blue circles) lupus patients at initial sample collection. (**B**) Volcano plot of differentially methylated CpG sites between African American (*n* = 22) and European American (*n* = 32) lupus patients at initial sample collection. Each dot represents a CpG site (*n* = 745,477). Significantly differentially methylated sites (green) are differentially methylated by at least 10% between ancestry groups and with an FDR-adjusted *P* < 0.05 (*n* = 907). (**C**) Pie chart (left) showing the percentage of sites hypermethylated (*n* = 420; 46.3%) and hypomethylated (*n* = 487; 53.7%) in African American compared with European American lupus patients. Barcharts showing the distribution of hypermethylated (red) and hypomethylated (blue) sites annotated to locations with CpG islands and genes (middle and right, respectively). S_Shore: south shore; S_Shelf: south shelf; N_Shore: north shore; N_Shelf: north shelf. 3′-UTR: 3′ untranslated region; ExonBnd: exon boundary; 5′-UTR: 5′ untranslated region; TSS200: 200 bp upstream of transcription start site; TSS1500: 1500 bp upstream of transcription start site.

We then performed a differential DNA methylation analysis comparing lupus patients with African American (*n* = 22) and European American (*n* = 32) ancestry after adjusting for medication use and age. A multidimensional plot of the 5000 most variable CpG sites in these patients showed that methylation patterns tended to cluster by patient ancestry group (Figure 2A). African American lupus patients in our cohort had more active disease compared with European American lupus patients (SLEDAI 5.2 ± 4.5 vs. 2.9 ± 3.2, respectively; *P* = 0.03; 2-tailed *t* test). Medication use at the initial time point was not significantly different between the ancestry groups (Supplemental Table 2). We identified 907 differentially methylated CpG sites using an FDR-adjusted *P* value threshold of < 0.05 and a differential methylation between ancestry groups of at least 10% (Figure 2B, Supplemental Table 3). Four hundred eighty-seven (53.7%) of these sites were hypomethylated in African American compared with European American lupus patients, and 420 (46.3%) were hypermethylated (Figure 2C). DNA methylation levels among differentially methylated sites differed by 16.5% on average (SD, 8.2%; range, 10.0%–57.9%) between ancestry groups. The hypomethylated and hypermethylated sites were associated with 391 and 316 genes, respectively. Hypomethylated genes showed enrichment for gene ontologies (GO) for granulocyte differentiation (GO: 0030852, FDR-adjusted *P* = 2.23 × 10^–2^; GO: 0030853, FDR-adjusted *P* = 3.20 × 10^–2^), cell adhesion (GO: 0007155, FDR-adjusted *P* = 1.26 × 10^–2^; GO: 1903037, FDR-adjusted *P* = 3.41 × 10^–2^), and TLR signaling pathways (GO: 0002224, FDR-adjusted *P* = 3.41 × 10^–2^; GO: 0034121, FDR-adjusted *P* = 4.46 × 10^–2^) (Supplemental Table 4). Hypermethylated genes were enriched for fewer ontologies representing primarily Rho guanine nucleotide exchange factor (GEF) protein activity (GO: 0005089, FDR-adjusted *P* = 7.76 × 10^–3^) and ATP binding (GO: 0005524, FDR-adjusted *P* = 2.80 × 10^–2^) (Supplemental Table 5).

To estimate the proportion of CpG sites differentially methylated between African American and European American lupus patients that are influenced by ancestral genetic differences, we performed differential DNA methylation analysis comparing neutrophils isolated from normal healthy African American and European American controls, using publicly available DNA methylation data generated using the Illumina HumanMethylation450 array (https://www.ncbi.nlm.nih.gov/geo/query/acc.cgi?acc=GSE65097). Of 437 sites differentially methylated between African American and European American lupus patients that were included on the HumanMethylation450 array, 185 CpG sites (42.3%) were also differentially methylated between African American and European American healthy controls (Supplemental Table 6). Indeed, among these methylation sites, DNA methylation differences between the 2 ancestries in controls and lupus patients showed a high degree of correlation (Pearson’s product-moment correlation *R* = 0.872; *t* statistic = 24.077, degrees of freedom [DF] = 183, *P* < 2.2 × 10^–16^). These data indicate that at least a proportion of differential methylation identified between African Americans and European American lupus patients can be explained by differences in the ancestral genetic background between the populations.

### meQTL analysis.

**Figure 3:**
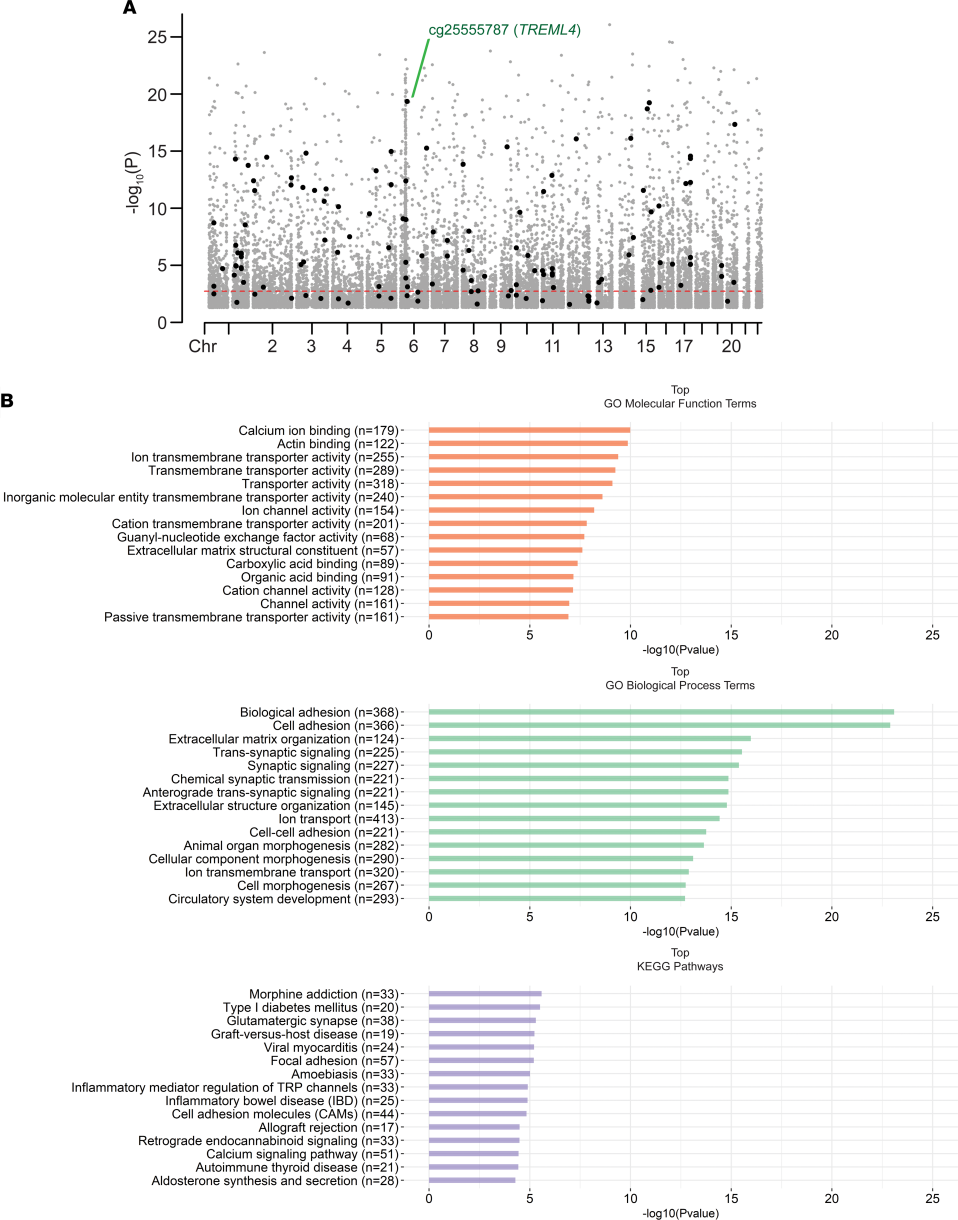
*cis*-meQTL analysis in lupus neutrophils. (**A**) A Manhattan plot showing CpG sites (black and gray dots) in *cis*-meQTL pairs identified in our lupus cohort. Black dots represent CpG sites in non–CpG-SNP *cis*-meQTL pairs that had a significantly different average methylation between African American and European American patients (FDR-adjusted *P* < 0.05). The red dashed line represents an approximate FDR-adjusted *P* value threshold of 0.05 for all *cis*-meQTL across the entire genome. An meQTL involving *TREML4* was among the most significant meQTL effects detected. (**B**) Enrichment of gene ontologies and pathways among annotated genes associated with CpG sites with *cis*-meQTL effects in lupus neutrophils. Barcharts show the most significant molecular function (orange) and biological process (green) gene ontology terms, and KEGG pathways (purple) by –log_10_ (*P* value). All terms have an FDR-adjusted *P* < 0.05.

**Figure 4:**
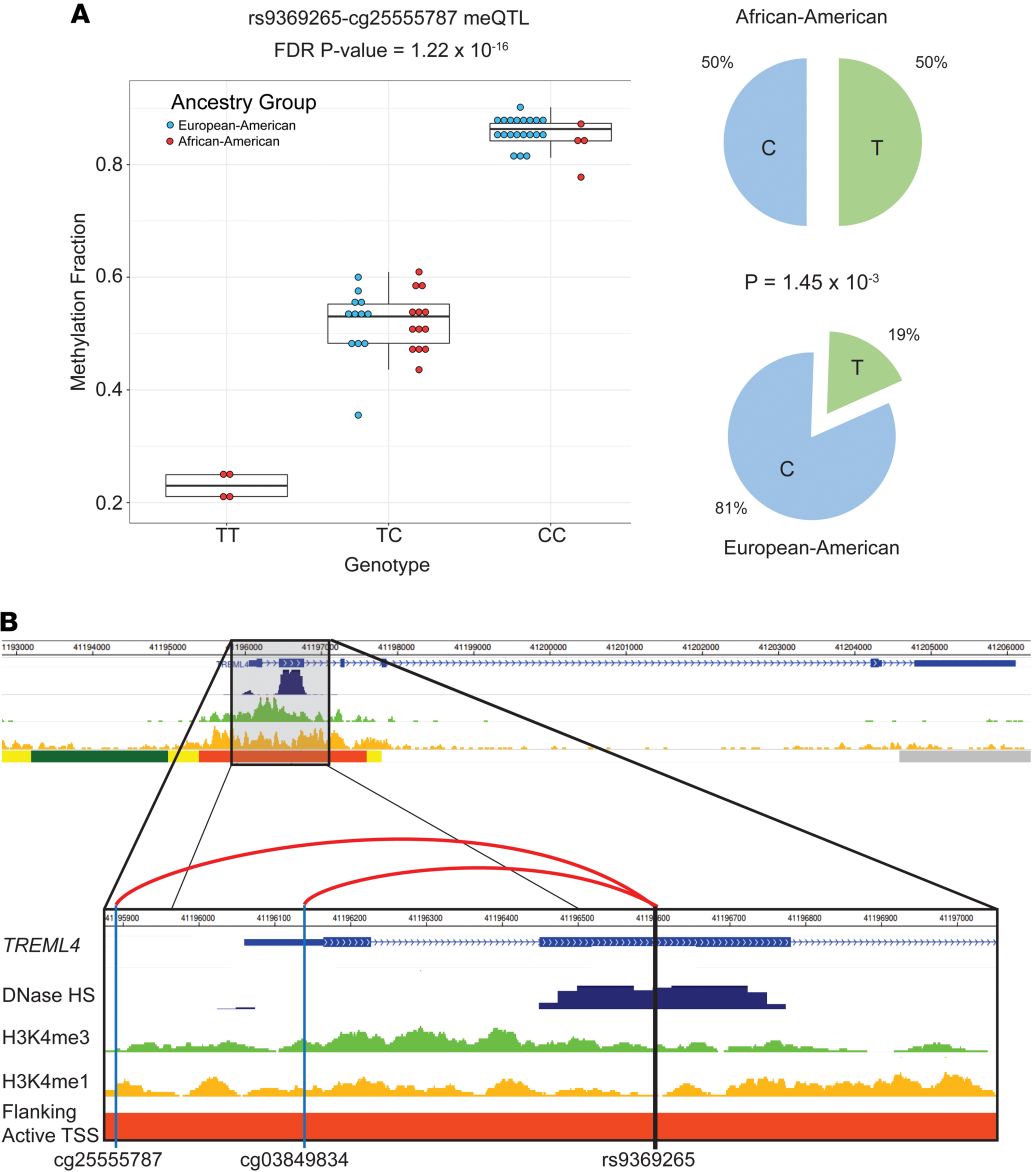
meQTL involving the SNP rs9369265 within *TREML4* in lupus patients. (**A**) Rs9369265 is significantly associated with the methylation status of cg25555787 (FDR-adjusted *P* value = 1.22 × 10^–16^). Mean β for genotype TT = 23% (25th percentile = 21.2%, 75th percentile = 25.0%, *n* = 4), mean β for genotype CT = 52.0% (25th percentile = 48.3%, 75th percentile = 55.2%, *n* = 25), and mean β for genotype CC = 85.6% (25th percentile = 84.2%, 75th percentile = 87.3%, *n* = 24). Whiskers extend to the maximum value within 1.5 times the IQR on either end of the group. Points beyond the whiskers are outliers. The minor allele frequency of rs9369265 significantly differed between European American (*n* = 32) and African American (*n* = 21) lupus patients (*P* = 1.45 × 10^–3^), with the T allele associated with lower DNA methylation. Comparing allelic proportions between ancestry groups was done using a 2-proportion *z* test. All *P* values were 2 tailed, and a significance threshold of *P* < 0.05 was used. (**B**) Rs9369265 is an exonic SNP in *TREML4* and is significantly associated with the methylation status of 2 CpG sites upstream of the transcription start site of *TREML4* (cg25555787 and cg03849834) (hg19). This region has epigenetic marks including DNase hypersensitivity (DNase HS), histone 3 lysine 4 mono- (H3K4me1) and –tri-methylation (H3K4me3) and is labeled as an enhancer region for *TREML4* (Flanking Active TSS; orange bar) in primary human neutrophils. Data for **B** were generated using the WashU Epigenome Browser (https://epigenomegateway.wustl.edu/) using ENCODE and Epigenome Roadmap ChromHMM data tracks from peripheral primary human neutrophils (E030).

We next identified associations between DNA methylation and genotype in our cross-sectional cohort of lupus patients (*n* = 53) after controlling for age, medications, and genetic background. *cis*-meQTL in our cohort (Figure 3A and Supplemental Table 7) were defined using a conservative range of 1000 bp to focus on localized effects. We identified a total of 8855 pairs of CpG sites and SNPs with an FDR-adjusted *P* < 0.05. These meQTL pairs represented 7614 (86.0%) unique methylation sites and 7094 (80.1%) unique polymorphisms. A total of 7269 (82.1%) of meQTL did not contain CpG-SNPs. Gene set enrichment analysis (GSEA) of the 3871 unique genes associated with the CpG sites revealed numerous ontologies and pathways. The most significantly enriched included ontologies and pathways for cell and biological adhesion (GO: 0007155, FDR-adjusted *P* = 6.43 × 10^–20^; GO: 0022610, FDR-adjusted *P* = 6.43 × 10^–20^; and KEGG: 83069, FDR-adjusted *P* = 5.29 × 10^–4^) and calcium ion binding and signaling pathways (GO: 0005509, FDR-adjusted *P* = 1.46 × 10^–7^; KEGG: 83050, FDR-adjusted *P* = 9.58 × 10^–4^) (Figure 3B and Supplemental Table 8). The meQTL revealed in our study are, at least in part, responsible for a proportion of the observed DNA methylation differences between African American and European American patients. Indeed, of the 907 differentially methylated CpG sites in our cohort, 142 (15.7%) were also meQTL (Figure 3A and Supplemental Table 9). These included sites associated with *IL16* (cg02810829; Δβ = –0.23) and an meQTL associated with the triggering receptor expressed on myeloid cells–like 4 (TREM-like 4) gene *TREML4* (cg25555787; Δβ = –0.20). Cg25555787 had one of the strongest meQTL associations in this study (rs9369265 meQTL, *R^2^* = 0.91) (Figure 4). We identified 1586 (17.9%) meQTL that were tagged as including CpG-SNPs and comprised many of the strongest methylation-genotype associations (Supplemental Table 7).

### meQTL involving lupus genetic susceptibility loci and type I IFN genes.

Comparing methylation site–associated genes in meQTL pairs with previously identified lupus susceptibility loci from genome-wide association studies (23–27), we identified 79 meQTL pairs in 28 lupus susceptibility genes (Supplemental Table 10). These included IFN regulatory factors *IRF7*, *IRF8*, and *STAT4*, which are involved in the type I IFN response. To identify type I IFN–regulated genes that are associated with meQTL in our cohort, we compared our meQTL-associated genes with the genes included in the Interferome (v.2.01) database (28). Sixty-four of the 3871 unique genes (1.7%) associated with methylation sites in meQTL were identified as type I IFN–regulated genes (Supplemental Table 11).

## Discussion

Neutrophils are the most numerous cells in circulating blood and are early responders to inflammatory events throughout the body. They play an important role in entering sites of infection to identify pathogens through a variety of receptors, destroying pathogens, and secreting inflammatory signals to mobilize the immune system in response (29). Their primary methods of destroying pathogens include phagocytosis, production of reactive oxygen species, release of granules containing antimicrobial enzymes, and the release of NETs, which physically bind and expose pathogens to antimicrobial proteins (29). In lupus, neutrophils display several abnormal phenotypes, including enhanced apoptosis, increased NETosis after type I IFN priming, and impaired phagocytosis (30). Our prior work has found that lupus neutrophils display a DNA methylation signature common to other immune cell types, primarily demethylation of type I IFN response genes (12).

We interrogated the DNA methylome of neutrophils in a cohort of lupus patients followed longitudinally for about 4 years across 229 time points to assess DNA methylation changes over time and across different levels of disease activity. We showed that the DNA methylome is largely stable over time and across disease activity in lupus patients. We identified 2 CpG sites (cg26104306 and cg06708913) with DNA methylation levels that significantly correlated with disease activity. These correlations were detected in African American but not European American lupus patients. Cg26104306 lies 745 bp upstream of the transcription start site of the gene *SNX18*, which encodes the sorting nexin 18 protein SNX18. It is located on the 5′ north shore of a CpG island (chr5:54517549–54519476 [hg38]) that overlaps the *SNX18* promoter region. Methylated CpG islands are typically indications of silenced gene promoters in somatic cells, and hypomethylation suggests disease-associated disruption in this silencing. SNX18 localizes to the plasma membrane of cells and plays a functional role in endocytosis and autophagosome formation in cells (31, 32). Cg06708913 overlapped a long noncoding RNA AC009522.1 and is proximal to an enhancer-like region denoted by transcriptionally permissive DNase hypersensitivity and increased H3K27ac modifications (33). One CpG site that reached suggestive significance for correlation with disease activity in African American lupus patients, cg24682077 (FDR-adjusted *P* = 5.35 × 10^–2^), is associated with FYVE, RhoGEF, and PH domain containing 1 gene *FGD1*. FGD1 interacts with Rho GTPase Cdc42, which regulates neutrophil motility in response to extracellular signals (34). Cg24682077 is located 39 bp downstream of the transcription start site of *FGD1* and within a promoter-associated CpG island.

The small number of CpG sites that change methylation levels with disease activity in our longitudinal study suggests that DNA methylation levels are stable in lupus neutrophils over time and across different disease activity levels. An inception study of lupus patients across time is necessary to detect DNA methylation biomarkers that indicate the onset of disease. A larger cohort size may bring more of these sites beyond the significance threshold or reveal novel associations in other ancestry groups. These associations will require replication to be confirmed but serve as indicators that novel disease–associated loci can be detected in longitudinal data from lupus patients. Our data also demonstrate that accounting for genetic ancestry in lupus studies can reveal novel associations.

Lupus nephritis is one of the most severe manifestation of lupus that can lead to chronic kidney damage and renal failure. We compared 2 time points from lupus patients with samples collected with and without nephritis in the same patient and adjusted DNA methylation changes for medication use, age, and race. A single methylation site, cg16204559, passed our FDR significance threshold corrected for multiple testing. Cg16204559 (chr11:11451256–11451258 [hg38]) is in the 11p15.4 cytoband within an intron of the gene *GALNT18*, which encodes the polypeptide N-acetylgalactosaminyltransferase 18 protein. 11p15.4 is adjacent to 11p15.5, which has previously been identified as the location of the Systemic Lupus Erythematosus Nephritis 3 (*SLEN3*) locus. This locus is near the short arm telomere of chromosome 11 and was identified as a susceptibility locus for lupus using genetic linkage in multiplexed pedigrees of African American ancestry that included lupus patients with nephritis (35). Understanding the biological role of this demethylation in lupus nephritis will require further investigation. Our study reveals the value of using longitudinal epigenetic studies to identify potentially novel DNA methylation changes that could provide insight for specific disease manifestations. Precision medicine approaches in lupus, enabling epigenetic modification in specific cell types and possibly in key specific genetic loci in the near future, are very promising (18).

Ancestry-associated DNA methylation differences and meQTL analyses showed a significant enrichment in Rho GEF pathways. GEFs are proteins that catalyze the cycling of GDP/GTP binding in Rho GTPases, which results in their activation (36, 37). Rho GTPase activity regulates neutrophil function by controlling cytoskeletal arrangements in response to activation of signaling pathways (38). They regulate reactive oxygen species production, endothelial adhesion and transmigration, and production of neutrophil extracellular traps (NETs) (38). We used network analysis to further characterize ancestry-specific differential DNA methylation in lupus. Supplemental Figure 1 shows a gene network that is centered around the transcription factor complex NF-κB. NF-κB is activated through degradation of inhibitory proteins in response to inflammatory signaling, such as TLR engagement, and translocates to the nucleus (39). There, it coordinates the expression of proinflammatory gene programs in neutrophils that delay apoptosis, promotes production of proinflammatory cytokines, and increases cell adhesion and NETosis when cells are sufficiently activated (40). Resting human neutrophils tightly regulate NF-κB activation through high levels of nuclear IκBα that is rapidly degraded upon proinflammatory stimulation (41). The gene *IKBKB* (cg20242624; Δβ = –0.11) encodes the inhibitor of NF-κB kinase subunit β protein (IKKβ), which is part of the IκB kinase (IKK) complex, required for activation and nuclear translocation of NF-κB by phosphorylation of the NF-κB inhibitory subunit IκBα (42). *BCL10* (cg17322118; Δβ = –0.18) encodes the B cell lymphoma/leukemia 10 protein BCL10, which is also an activator of NF-κB through ubiquitination of the IKK subunit protein IKKγ (43). Hypomethylation of these genes in neutrophils may reflect an increased response to inflammatory stimuli that promotes tissue invasion and inflammatory damage. Differentially methylated genes involved in regulating the type I IFN response were also present (Supplemental Figure 2). In particular *IRF7* (cg08926253, Δβ = –0.14; cg22016995, Δβ = 0.13) was significantly hypomethylated, similar to what we previously observed in naive CD4^+^ T cells of African American lupus patients and the neutrophils of lupus patients compared with healthy controls (12, 44). *IFI44*, also a type I IFN response gene, was also significantly hypomethylated in African American patients (cg01079652; Δβ = –0.23). We compared differentially hypomethylated genes in African American lupus patients to the Interferome (v.2.01) database (28) to identify other type I IFN–regulated genes (Supplemental Table 11). Of interest, the cytokine gene *IL16* was hypomethylated in African American patients (cg02810829; Δβ = –0.23) and had a modest association in an meQTL pair (rs35130261 meQTL R^2^ = 0.68; Δ minor allele frequency [ΔMAF] = 0.33). IL-16 is a chemoattractant cytokine that induces infiltration of T cells, macrophages, and eosinophils into sites of inflammation and promotes proinflammatory cytokine release by monocytes in vitro (45). It also promotes IL-2 receptor expression on the surface of CD4^+^ T cells, enhancing IL-2 activity (46) and the migration and expansion of Tregs in sites of inflammation (47). A recent study observed that neutrophils produce and store inactive pro–IL-16 in the cytosol, which is released and activated by caspase-3 upon secondary necrosis (48). Increased circulating IL-16 levels in lupus patients are associated with more severe disease (49, 50), and primary neutrophils of lupus patients more readily undergo apoptosis and increased secondary necrosis with reduced clearance of apoptotic material (51). This suggests that hypomethylation of *IL16* (in part, related to meQTL) could promote an exaggerated inflammatory response upon neutrophil secondary necrosis in lupus patients.

A demonstration of the mechanism underlying meQTL associations can be seen in 2 of the strongest meQTL pairs, cg25555787 (*TREML4*; rs9369265 meQTL *R^2^* = 0.91; ΔMAF = 0.31) and cg03849834 (*TREML4*; rs9369265 meQTL *R^2^* = 0.81; ΔMAF = 0.31). Functionally, TREML4 has previously been identified as playing an important role in modulating the response to TLR7 signaling when bound to single-stranded RNA and TLR9 binding to unmethylated CpG-DNA (52). Rs9369265 lies in the second exon of *TREML4* within an active region flanking the transcription starts site of *TREML4* in neutrophils. Rs9369265 genotype is also significantly associated with the expression of *TREML4* in whole blood (Gene-Tissue Expression Portal [GTEx] *P* = 1.2 × 10^–163^), with the C allele associated with reduced expression and increased DNA methylation in our data. The presence of H3K4me3 peaks in this region and DNase accessibility suggest this is an important regulatory region for controlling *TREML4* expression as a promoter. A reduction in DNA methylation corresponds with an increase in H3K4me3 and promoter activity (53). The ligand for *TREML4* is unknown, but it readily binds to dead and dying cells (54). Reduced clearance of necrotic material in lupus patients might provide more stimulation to TREML4 and TLRs, promoting the exaggerated type I IFN response seen in lupus patients and contributing to the development of renal disease in lupus (55). Indeed, it has been observed that lupus-prone MRL*/lpr* mice have higher survival, produce fewer dsDNA autoantibodies, and develop less renal damage when *Treml4* is knocked out (52). Neutrophils from *Treml4^–/–^* mice show reduced expression of *Cxcl2*, which is a potent neutrophil chemoattractant, but unimpaired motility and phagocytosis (52). The higher frequency of the T allele in our African American lupus patients, which correlates with increased *TREML4* expression, suggests a potential for a more robust response to TLR stimulation. This is supported by the observation of increased expression of the proinflammatory cytokines IFN-α and TNF-α in the whole blood of female African American lupus patients compared with female European American patients (56).

The mechanisms underlying the association between genotype and DNA methylation status will require further investigation. Potential mechanisms could include an inherited haplotype tagged by rs9369265 that promotes or suppresses transcription regulator accessibility and binding. This effect could also extend to other myeloid cells that express *TREML4*, including macrophages and DCs, which contribute to the proinflammatory response (52).

We compared the DNA methylation profiles of neutrophils from a small cohort of healthy African American and European American female controls to determine if the observed differences in lupus patients were unique to the disease or shared by healthy ancestral populations. Of methylation sites that were assessed in both patients and healthy controls, 42.3% of the differentially methylated sites between African American and European American lupus patients overlapped with differentially methylated sites in healthy control neutrophils between the 2 populations. Furthermore, 30 (48.4%) of the 62 CpG sites included on both the Infinium MethylationEPIC and Infinium HumanMethylation450 arrays that are in an meQTL pair and differentially methylated in patient neutrophils were also differentially methylated in control neutrophils, between the 2 ancestries. Taken together, ancestry-associated methylation variability in lupus patients includes both genetically determined methylation differences and methylation changes that might be related to nongenetic factors. Additional work is required to differentiate benign ancestry-associated epigenetic variability from epigenetic changes that might contribute to the pathogenesis of lupus or to differences in disease presentation and progression between populations.

Our current study was focused on epigenetic evaluation of neutrophils isolated from lupus patients, given the increasingly recognized role of neutrophil dysfunction in lupus. Future longitudinal studies in other cell types involved in the pathogenesis of lupus are likely to provide additional insights. For example, a prominent role for T cell aberrancies in the pathogenesis of lupus is well established (57). Investigating T cell DNA methylation changes over time in patients with lupus is warranted.

In summary, we have analyzed the association of DNA methylation with disease activity across time in the neutrophils of lupus patients. We demonstrate that the DNA methylome is at least in part determined by genetic variants in lupus patients and is largely stable over time and across disease activity levels in a longitudinal multiancestral lupus cohort. We identified 2 CpG sites in patients of African American ancestry that show methylation levels associated with disease activity in lupus. We also identified a single locus that becomes hypomethylated in patients who developed active lupus nephritis. Using genome-wide DNA methylation and genotyping data, we characterized ancestry-associated DNA methylation changes in lupus neutrophils and identified meQTL effects throughout the genome. Two genes, *TREML4* and *IL16*, contained meQTL and were also significantly hypomethylated in African American lupus patients. These genes play roles in promoting inflammatory response to TLR signaling and infiltration of peripheral immune cells into tissue.

## Methods

### Study participants and demographics.

Fifty-four female lupus patients were recruited from the University of Michigan Health System and Henry Ford Health System for this study (Supplemental Table 2). Our cohort included 32 patients of European American ancestry and 22 patients of African American ancestry. Patients were followed over a 43-month period. The patients selected for this study had at least 1 change in disease activity as measured by the SLEDAI score across all time points. This resulted in a total of 229 time points across all patients (4 median time points per patient; range, 2–11 time points). The mean age of patients at the initial visit was 41.0 ± 13.1 years (mean ± SD; range, 19–70 years). The mean SLEDAI score of patients was 3.9 ± 3.9 (mean ± SD; range, 0–20) at their initial visit and 4.0 ± 3.7 (mean ± SD; range, 0–20) across all time points (Supplemental Figure 3). All patients in this study fulfilled the American College of Rheumatology classification criteria for SLE (58).

DNA methylation data from normal healthy control neutrophils (*n* = 5 and 6 African American and European American, respectively) generated using the Illumina Infinium HumanMethylation450 array and previously reported were also used (12) (GEO accession no. GSE65097; https://www.ncbi.nlm.nih.gov/geo/query/acc.cgi?acc=GSE65097). DNA methylation data generated in this study have been deposited in Gene Expression Omnibus (GEO) and are available under GEO accession number GSE161476.

### DNA isolation.

Whole blood was collected from each patient at each time point during clinic visits in vials containing EDTA. Granulocyte fractions were isolated using density centrifugation with Ficoll-Histopaque (GE Healthcare). Genomic DNA was isolated from the enriched granulocyte layer using either phenol-chloroform extraction or QIAGEN DNEasy Blood and Tissue kit (QIAGEN), or following the removal of RBCs using dextran (MilliporeSigma) and hypotonic lysis (59). DNA was eluted in water and quantified using Qubit DNA fluorescence quantification assays (Thermo Fisher Scientific).

### DNA methylation measurement.

A total of 350ng of DNA from each sample was bisulfite converted using the EZ-96 DNA Methylation Kit (Zymo) following the manufacturer’s instructions. Samples were hybridized to the Infinium MethylationEPIC array (Illumina) to assess site-specific DNA methylation of over 850,000 methylation sites across the genome. Samples were randomized across all arrays to minimize batch effects. Sample hybridization and array scanning were performed at the University of Michigan Advanced Genomics Core.

### DNA methylation quality control and analysis.

DNA methylation data analysis was performed in the R statistical computing environment (v.3.6.3) (60). Raw.idat files were generated for each sample and read into the R package *minfi* (v.1.32.0) for quality control (QC) and downstream analysis (61, 62). Probes with fewer than 3 beads and zero intensity values across all samples were removed according to best practices as implemented by the *DNAmArray* package (v.0.1.1) (63). Then, background signal and dye bias were corrected, followed by normalization of signal intensities using functional normalization in the *preprocessFunnorm.DNAmArray* function (63, 64). This method uses the first 3 principal component values calculated from signal intensities of control probes present on all array spots to correct for technical variation. Probes with detection *P* < 0.01 were removed, as were probes that returned signal intensities in fewer than 98% of samples. Signal intensities were then converted to M-values with a maximum bound of ± 16. M-values were used for all regression testing and converted to β values (0%–100% methylation scale) using *minfi* for reporting.

We masked any probes with potential technical issues if the probe met any one of the following criteria described by ref. 65: A unique probe sequence of less than 30 bp, mapping to multiple sites in the genome, polymorphisms that cause a color channel switching in type I probes, inconsistencies in specified reporter color channel and extension base, mapping to the Y chromosome, and/or having a polymorphism within 5 bp of the 3′ end of the probe with a MAF > 1% with exception of CpG-SNPs with C > T polymorphisms, which we retained for analysis. Batch correction was performed using the *ComBat* function in the *sva* (v.3.34.0) package (66).

We implemented a mixed correspondence analysis with the *PCAmixdata* package (v.3.1) to calculate eigenvalues using patient medication data for prednisone, hydroxychloroquine, azathioprine, mycophenolate mofetil, and cyclophosphamide (67). The top 4 components accounted for a cumulative 88.4% of variability in the medication data. Each component value was used as an independent variable in regression analysis to adjust for medication usage across individuals.

Cell type–specific DNA methylation profiles were used to assess enrichment of neutrophils in our DNA samples (68). Of 73 CpG sites previously identified to accurately discriminate between neutrophils and other cell types in peripheral blood (namely CD4^+^ T cells, CD8^+^ T cells, B cells, NK cells, and monocytes), methylation levels in 71 sites passed our QC measures in our data set. DNA methylation levels in these sites were very highly correlated in our DNA samples with DNA isolated from neutrophils (*R* = 0.996, Supplemental Figure 4).

### Genotyping and meQTL analysis.

Genotyping data were generated using Infinium Global Screening Array-24 v2.0 (Illumina) according to the manufacturer’s instructions. Stringent QCs were applied before analyses using *PLINK* (v.1.9) (69). Single nucleotide polymorphisms (SNPs) with a genotyping call rate < 98% and MAF < 5%, as well as those showing a deviation from Hardy-Weinberg equilibrium (HWE; *P* < 1 × 10^–3^), were filtered out. Samples were removed if they had a genotyping call rate < 95%. Sex chromosomes were not analyzed. About 100,000 independent SNPs were pruned and used to perform principal component analysis (PCA) with *Eigensoft* (v.6.1.4) software (Supplemental Figure 5) (70). Genotyping data of a single African American lupus patient were removed at the QC step due to failing quality measures. All meQTL analyses presented in this paper are obtained from the methylation and genotyping profiles of *n* = 21 African American and *n* = 32 European American lupus patients.

### GSEA.

Gene annotation of CpG probes was done using GENCODE v22 (hg38) annotations from a manifest file produced by ref. 65. Gene network analysis of differentially methylated genes was done using Ingenuity Pathway Analysis (QIAGEN; https://analysis.ingenuity.com/). ToppGene Suite was used for functional gene ontology enrichment analysis (71). Molecular Function and Biological Process Gene Ontologies and KEGG Pathways were selected for enrichment. *P* values were derived using a hypergeometric probability mass function and a Benjamini-Hochberg FDR–adjusted *P* value cutoff of < 0.05 was used as a threshold of significance. Ontologies and pathways had to have a minimum membership of 3 genes and maximum of 2000 genes to be included.

IFN-regulated genes were identified using the differentially methylated gene set as input for Interferome (v.2.01) (28), limiting results to genes with an expression fold change of 1.5 or greater between type I IFN–treated and untreated samples using data sets derived from peripheral whole blood.

### Statistics.

We used probe-wise linear regressions to detect CpG sites in our cohort that show methylation difference between African American and European American patients using the *limma* (v.3.42.2) package (72). Patient age and the top 4 medication components were adjusted for in each regression, and an empirical Bayes moderated t-statistic and *P* value calculated for each probe. CpG sites were considered to be significantly differentially methylated if they had a Benjamini-Hochberg FDR-adjusted *P* < 0.05 and were differentially methylated by at least 10% between African American and European American patients.

Methylation M-values from the initial time point samples (*n* = 53), sample genotypes (*n* = 53), sample age, the top 4 medication components, and top 10 genotype principal components were used to build a linear model for detecting meQTL using *MatrixEQTL* (v.2.3) in R (73). *cis*-meQTL were defined as CpG sites with methylation values associated with a SNP within a conservative 1000 bp of the CpG dinucleotide. We used a Benjamini-Hochberg FDR-adjusted *P* value cutoff of < 0.05 for significant associations.

Analysis of the association with SLEDAI score and DNA methylation in our longitudinal cohort (*n* = 93 African American and 136 European American patient samples) was performed by fitting a linear mixed model using the *lmerTest* (v.3.1-2) (74) and *MuMIn* (v.1.43.17) packages in R (75). A regression model was fit in a probe-wise manner for all samples in each ancestry group. Regression models were adjusted for age at sample collection as a fixed effect and SLEDAI score as the variable of interest. Repeated samples were grouped by patient, which was accounted for as a random effect in the model. CpG methylation and SLEDAI score had a statistically significant association if they had a Benjamini-Hochberg FDR-adjusted *P* < 0.05 and a suggestive association with a Benjamini-Hochberg FDR-adjusted *P* < 0.10. The impact of adjusting for medication components was determined by comparing the fit of the previously specified mixed effect regression model with an extended model that includes additional fixed effects for the top 4 medication components of each lupus patient’s time point. A χ^2^ difference test for nested models was applied using the ANOVA function in R to determine if model fit was improved. *P* < 0.05 was considered statistically significant and indicates that the larger model has improved data fit.

Longitudinal analysis of nephritis in our cohort was performed by fitting a linear mixed model as above to each probe using methylation profiles for *n* = 11 lupus patients (*n* = 7 African American and *n* = 4 European American) at a time point with active nephritis (as defined by SLEDAI) and the nearest preceding or receding time point without nephritis after adjusting for the top 4 medication components, age, and ancestry as fixed effects. Sample pairs were included as a random effect. A Benjamini-Hochberg FDR-adjusted *P* value threshold of < 0.05 was used to identify statistically significant associations.

Two-group testing of mean ages between ancestry groups was done using a 2-tailed *t* test. SLEDAI criteria and medication differences were compared using Fisher’s exact test. Comparing allelic proportions between ancestry groups was done using a 2-proportion *z* test. All *P* values were 2-tailed and a significance threshold of *P* < 0.05 was used.

### Study approval.

The IRBs of the University of Michigan Health System, Henry Ford Health System, and the University of Pittsburgh approved this study, and all patients signed informed consent before study enrollment.

## Author contributions

PC designed and performed the experiments, analyzed the data, and wrote the manuscript. LOF analyzed genotyping data. EEL, WJM, and KMM recruited lupus patients, provided samples, and assessed lupus disease activity. AHS conceived and designed the study and contributed to data analysis and writing of the manuscript. All authors critically reviewed and edited the manuscript and approved submission.

## Supplementary Material

Supplemental data

Supplemental Table 1

## Figures and Tables

**Table 1:**
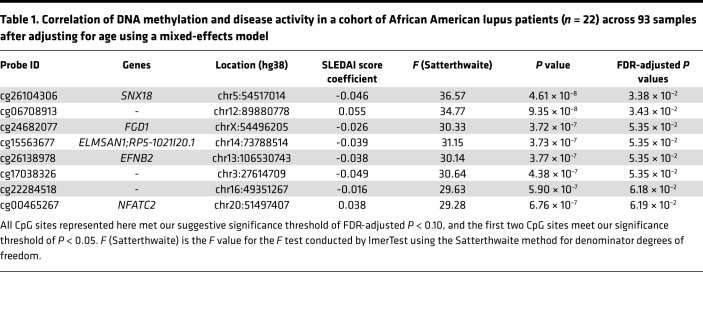
Correlation of DNA methylation and disease activity in a cohort of African American lupus patients (*n* = 22) across 93 samples after adjusting for age using a mixed-effects model

## Version 1 10/27/2020

In-Press Preview

## Version 2 11/19/2020

Electronic publication

## References

[1] Tsokos GC (2020). Autoimmunity and organ damage in systemic lupus erythematosus. Nat Immunol.

[2] Györi N, Giannakou I, Chatzidionysiou K, Magder L, van Vollenhoven RF, Petri M (2017). Disease activity patterns over time in patients with SLE: analysis of the Hopkins Lupus Cohort. Lupus Sci Med.

[3] Alarcón GS, Ugarte-Gil MF, Pons-Estel G, Vilá LM, Reveille JD, McGwin G (2019). Remission and low disease activity state (LDAS) are protective of intermediate and long-term outcomes in SLE patients. Results from LUMINA (LXXVIII), a multiethnic, multicenter US cohort. Lupus.

[4] Baechler EC (2003). Interferon-inducible gene expression signature in peripheral blood cells of patients with severe lupus. Proc Natl Acad Sci U S A.

[5] Han GM, Chen SL, Shen N, Ye S, Bao CD, Gu YY (2003). Analysis of gene expression profiles in human systemic lupus erythematosus using oligonucleotide microarray. Genes Immun.

[6] Bennett L (2003). Interferon and granulopoiesis signatures in systemic lupus erythematosus blood. J Exp Med.

[7] Banchereau R (2016). Personalized Immunomonitoring Uncovers Molecular Networks that Stratify Lupus Patients. Cell.

[8] Mistry P (2019). Transcriptomic, epigenetic, and functional analyses implicate neutrophil diversity in the pathogenesis of systemic lupus erythematosus. Proc Natl Acad Sci U S A.

[9] Teruel M, Sawalha AH (2017). Epigenetic Variability in Systemic Lupus Erythematosus: What We Learned from Genome-Wide DNA Methylation Studies. Curr Rheumatol Rep.

[10] Coit P (2016). Epigenetic Reprogramming in Naive CD4+ T Cells Favoring T Cell Activation and Non-Th1 Effector T Cell Immune Response as an Early Event in Lupus Flares. Arthritis Rheumatol.

[11] Coit P (2015). Renal involvement in lupus is characterized by unique DNA methylation changes in naïve CD4+ T cells. J Autoimmun.

[12] Coit P (2015). Epigenome profiling reveals significant DNA demethylation of interferon signature genes in lupus neutrophils. J Autoimmun.

[13] Imgenberg-Kreuz J (2018). DNA methylation mapping identifies gene regulatory effects in patients with systemic lupus erythematosus. Ann Rheum Dis.

[14] Lanata CM (2019). A phenotypic and genomics approach in a multi-ethnic cohort to subtype systemic lupus erythematosus. Nat Commun.

[15] Koelsch KA (2013). Functional characterization of the MECP2/IRAK1 lupus risk haplotype in human T cells and a human MECP2 transgenic mouse. J Autoimmun.

[16] González LA, Toloza SM, Alarcón GS (2014). Impact of race and ethnicity in the course and outcome of systemic lupus erythematosus. Rheum Dis Clin North Am.

[17] Rohraff DM, He Y, Farkash EA, Schonfeld M, Tsou PS, Sawalha AH (2019). Inhibition of EZH2 Ameliorates Lupus-Like Disease in MRL/lpr Mice. Arthritis Rheumatol.

[18] Li H (2018). Precision DNA demethylation ameliorates disease in lupus-prone mice. JCI Insight.

[19] Zhao M (2016). IFI44L promoter methylation as a blood biomarker for systemic lupus erythematosus. Ann Rheum Dis.

[20] Renauer P (2015). DNA methylation patterns in naïve CD4+ T cells identify epigenetic susceptibility loci for malar rash and discoid rash in systemic lupus erythematosus. Lupus Sci Med.

[21] Ballestar E, Sawalha AH, Lu Q (2020). Clinical value of DNA methylation markers in autoimmune rheumatic diseases. Nat Rev Rheumatol.

[22] Pidsley R (2016). Critical evaluation of the Illumina MethylationEPIC BeadChip microarray for whole-genome DNA methylation profiling. Genome Biol.

[23] Morris DL (2016). Genome-wide association meta-analysis in Chinese and European individuals identifies ten new loci associated with systemic lupus erythematosus. Nat Genet.

[24] Bentham J (2015). Genetic association analyses implicate aberrant regulation of innate and adaptive immunity genes in the pathogenesis of systemic lupus erythematosus. Nat Genet.

[25] Han JW (2009). Genome-wide association study in a Chinese Han population identifies nine new susceptibility loci for systemic lupus erythematosus. Nat Genet.

[26] Alarcón-Riquelme ME (2016). Genome-Wide Association Study in an Amerindian Ancestry Population Reveals Novel Systemic Lupus Erythematosus Risk Loci and the Role of European Admixture. Arthritis Rheumatol.

[27] Lee YH, Bae SC, Choi SJ, Ji JD, Song GG (2012). Genome-wide pathway analysis of genome-wide association studies on systemic lupus erythematosus and rheumatoid arthritis. Mol Biol Rep.

[28] Rusinova I (2013). Interferome v2.0: an updated database of annotated interferon-regulated genes. Nucleic Acids Res.

[29] Amulic B, Cazalet C, Hayes GL, Metzler KD, Zychlinsky A (2012). Neutrophil function: from mechanisms to disease. Annu Rev Immunol.

[30] Kaplan MJ (2013). Role of neutrophils in systemic autoimmune diseases. Arthritis Res Ther.

[31] Park J (2010). SNX18 shares a redundant role with SNX9 and modulates endocytic trafficking at the plasma membrane. J Cell Sci.

[32] Knævelsrud H (2013). Membrane remodeling by the PX-BAR protein SNX18 promotes autophagosome formation. J Cell Biol.

[33] ENCODE Project Consortium (2012). An integrated encyclopedia of DNA elements in the human genome. Nature.

[34] Szczur K, Xu H, Atkinson S, Zheng Y, Filippi MD (2006). Rho GTPase CDC42 regulates directionality and random movement via distinct MAPK pathways in neutrophils. Blood.

[35] Quintero-Del-Rio AI, Kelly JA, Kilpatrick J, James JA, Harley JB (2002). The genetics of systemic lupus erythematosus stratified by renal disease: linkage at 10q22.3 (SLEN1), 2q34-35 (SLEN2), and 11p15.6 (SLEN3). Genes Immun.

[36] Rossman KL, Der CJ, Sondek J (2005). GEF means go: turning on RHO GTPases with guanine nucleotide-exchange factors. Nat Rev Mol Cell Biol.

[37] Deaton AM, Bird A (2011). CpG islands and the regulation of transcription. Genes Dev.

[38] Pantarelli C, Welch HCE (2018). Rac-GTPases and Rac-GEFs in neutrophil adhesion, migration and recruitment. Eur J Clin Invest.

[39] Sabroe I, Dower SK, Whyte MK (2005). The role of Toll-like receptors in the regulation of neutrophil migration, activation, and apoptosis. Clin Infect Dis.

[40] Mussbacher M (2019). Cell Type-Specific Roles of NF-κB Linking Inflammation and Thrombosis. Front Immunol.

[41] Castro-Alcaraz S, Miskolci V, Kalasapudi B, Davidson D, Vancurova I (2002). NF-kappa B regulation in human neutrophils by nuclear I kappa B alpha: correlation to apoptosis. J Immunol.

[42] Israël A (2010). The IKK complex, a central regulator of NF-kappaB activation. Cold Spring Harb Perspect Biol.

[43] Zhou H (2004). Bcl10 activates the NF-kappaB pathway through ubiquitination of NEMO. Nature.

[44] Coit P, Ognenovski M, Gensterblum E, Maksimowicz-McKinnon K, Wren JD, Sawalha AH (2015). Ethnicity-specific epigenetic variation in naïve CD4+ T cells and the susceptibility to autoimmunity. Epigenetics Chromatin.

[45] Mathy NL (2000). Interleukin-16 stimulates the expression and production of pro-inflammatory cytokines by human monocytes. Immunology.

[46] Parada NA (1998). Synergistic activation of CD4+ T cells by IL-16 and IL-2. J Immunol.

[47] McFadden C (2007). Preferential migration of T regulatory cells induced by IL-16. J Immunol.

[48] Roth S (2015). Secondary necrotic neutrophils release interleukin-16C and macrophage migration inhibitory factor from stores in the cytosol. Cell Death Discov.

[49] Lard LR, Roep BO, Verburgh CA, Zwinderman AH, Huizinga TW (2002). Elevated IL-16 levels in patients with systemic lupus erythematosus are associated with disease severity but not with genetic susceptibility to lupus. Lupus.

[50] Lee S, Kaneko H, Sekigawa I, Tokano Y, Takasaki Y, Hashimoto H (1998). Circulating interleukin-16 in systemic lupus erythematosus. Br J Rheumatol.

[51] Mahajan A, Herrmann M, Muñoz LE (2016). Clearance Deficiency and Cell Death Pathways: A Model for the Pathogenesis of SLE. Front Immunol.

[52] Ramirez-Ortiz ZG (2015). The receptor TREML4 amplifies TLR7-mediated signaling during antiviral responses and autoimmunity. Nat Immunol.

[53] Sharifi-Zarchi A (2017). DNA methylation regulates discrimination of enhancers from promoters through a H3K4me1-H3K4me3 seesaw mechanism. BMC Genomics.

[54] Hemmi H (2009). A new triggering receptor expressed on myeloid cells (Trem) family member, Trem-like 4, binds to dead cells and is a DNAX activation protein 12-linked marker for subsets of mouse macrophages and dendritic cells. J Immunol.

[55] Devarapu SK, Anders HJ (2018). Toll-like receptors in lupus nephritis. J Biomed Sci.

[56] Lyn-Cook BD (2014). Increased expression of Toll-like receptors (TLRs) 7 and 9 and other cytokines in systemic lupus erythematosus (SLE) patients: ethnic differences and potential new targets for therapeutic drugs. Mol Immunol.

[57] Moulton VR, Tsokos GC (2015). T cell signaling abnormalities contribute to aberrant immune cell function and autoimmunity. J Clin Invest.

[58] Hochberg MC (1997). Updating the American College of Rheumatology revised criteria for the classification of systemic lupus erythematosus. Arthritis Rheum.

[59] Clark RA, Nauseef WM (2001). Isolation and functional analysis of neutrophils. Curr Protoc Immunol.

[60] Team R. R: A Language Environment for Statistical Computing. R Project. https://www.r-project.org/ Accessed October 20, 2020

[61] Aryee MJ (2014). Minfi: a flexible and comprehensive Bioconductor package for the analysis of Infinium DNA methylation microarrays. Bioinformatics.

[62] Fortin JP, Triche TJ, Hansen KD (2017). Preprocessing, normalization and integration of the Illumina HumanMethylationEPIC array with minfi. Bioinformatics.

[63] Sinke L, et al. DNAmArray: Streamlined workflow for the quality control, normalization, analysis of Illumina methylation array data. Zenodo. https://zenodo.org/record/3355292 Published July 11, 2019. Accessed October 20, 2020

[64] Fortin JP (2014). Functional normalization of 450k methylation array data improves replication in large cancer studies. Genome Biol.

[65] Zhou W, Laird PW, Shen H (2017). Comprehensive characterization, annotation and innovative use of Infinium DNA methylation BeadChip probes. Nucleic Acids Res.

[66] Leek JT, Johnson WE, Parker HS, Jaffe AE, Storey JD (2012). The sva package for removing batch effects and other unwanted variation in high-throughput experiments. Bioinformatics.

[67] Chavent M, Kuentz-Simonet V, Labenne A, Saracco J. Multivariate analysis of mixed data: The R Package PCAmixdata. Cornell University. https://arxiv.org/abs/1411.4911 Updated December 8, 2017. Accessed October 20, 2020

[68] Salas LA (2018). An optimized library for reference-based deconvolution of whole-blood biospecimens assayed using the Illumina HumanMethylationEPIC BeadArray. Genome Biol.

[69] Chang CC, Chow CC, Tellier LC, Vattikuti S, Purcell SM, Lee JJ (2015). Second-generation PLINK: rising to the challenge of larger and richer datasets. Gigascience.

[70] Price AL, Patterson NJ, Plenge RM, Weinblatt ME, Shadick NA, Reich D (2006). Principal components analysis corrects for stratification in genome-wide association studies. Nat Genet.

[71] Chen J, Bardes EE, Aronow BJ, Jegga AG (2009). ToppGene Suite for gene list enrichment analysis and candidate gene prioritization. Nucleic Acids Res.

[72] Ritchie ME (2015). limma powers differential expression analyses for RNA-sequencing and microarray studies. Nucleic Acids Res.

[73] Shabalin AA (2012). Matrix eQTL: ultra fast eQTL analysis via large matrix operations. Bioinformatics.

[74] Kuznetsova A, Brockhoff PB, Christensen RHB (2017). lmerTest Package: Tests in Linear Mixed Effects Models. J Stat Softw.

[75] Barton K. MuMIn: Multi-Model Inference. R Project. https://cran.r-project.org/web/packages/MuMIn/MuMIn.pdf Published April 14, 2020. Accessed October 20, 2020

