# Supplementary material for: A longitudinal and transancestral analysis of DNA methylation patterns and disease activity in lupus patients
Source: JCI Insight. 2020 Nov 19;5(22):e143654. doi: 10.1172/jci.insight.143654 (PMC7710270; doi:10.1172/jci.insight.143654)
Supplement: Supplemental data [file jciinsight-5-143654-s201.pdf]

Supplemental Figure 1. Networks of genes with hypomethylated (green shapes) and hypermethylated (red shapes) CpG sites in African-American compared to European-American lupus patients. White shapes represent genes included in the network by IPA through their relationship to the input genes. Green-red shapes included both hypo- and hypermethylated CpG sites. Dashed and solid lines represent indirect and direct interactions, respectively.

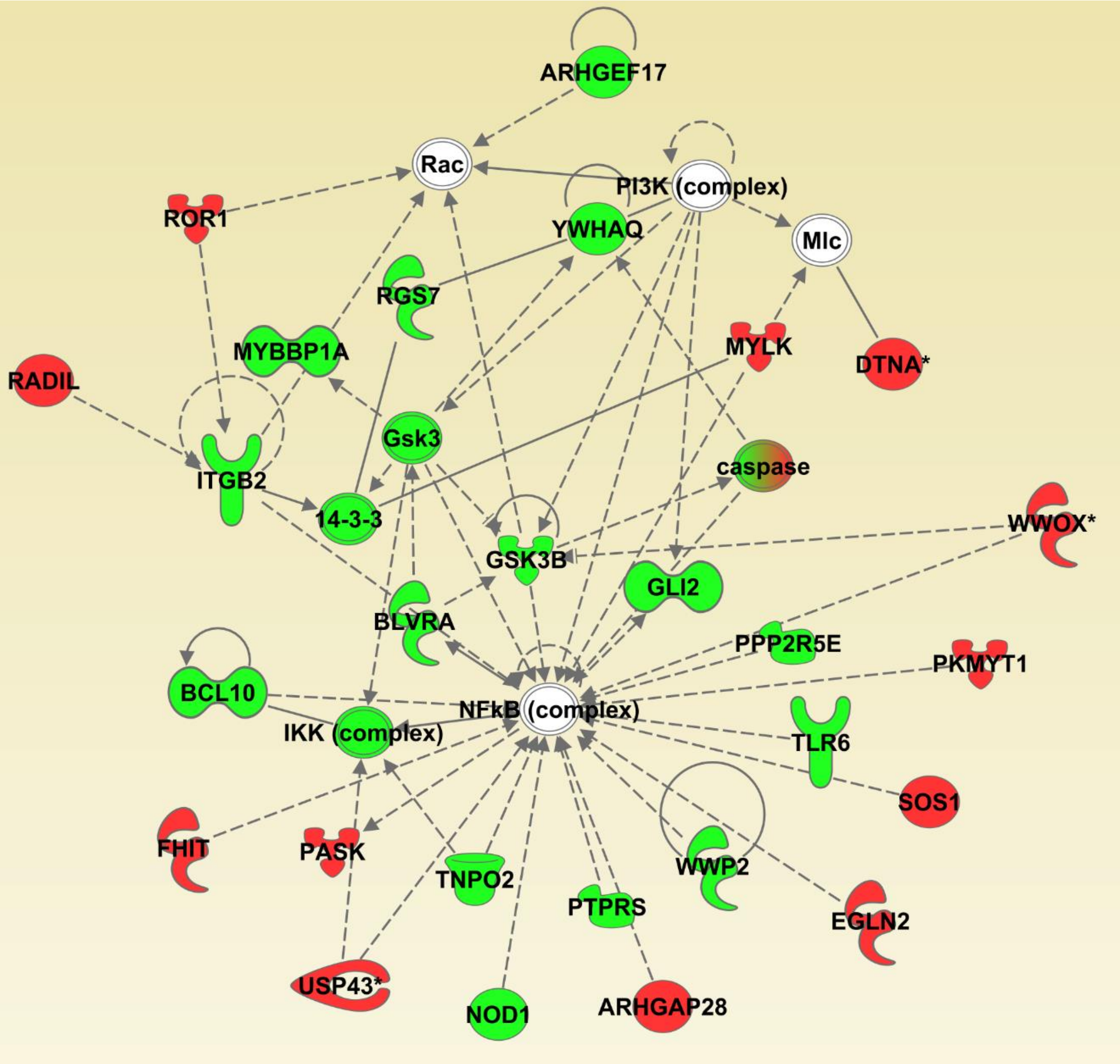

Supplemental Figure 2: Networks of genes with hypomethylated (green shapes) and hypermethylated (red shapes) CpG sites in African-American compared to European-American lupus patients. White shapes represent genes included in the network by IPA through their relationship to the input genes. Dashed and solid lines represent indirect and direct interactions, respectively.

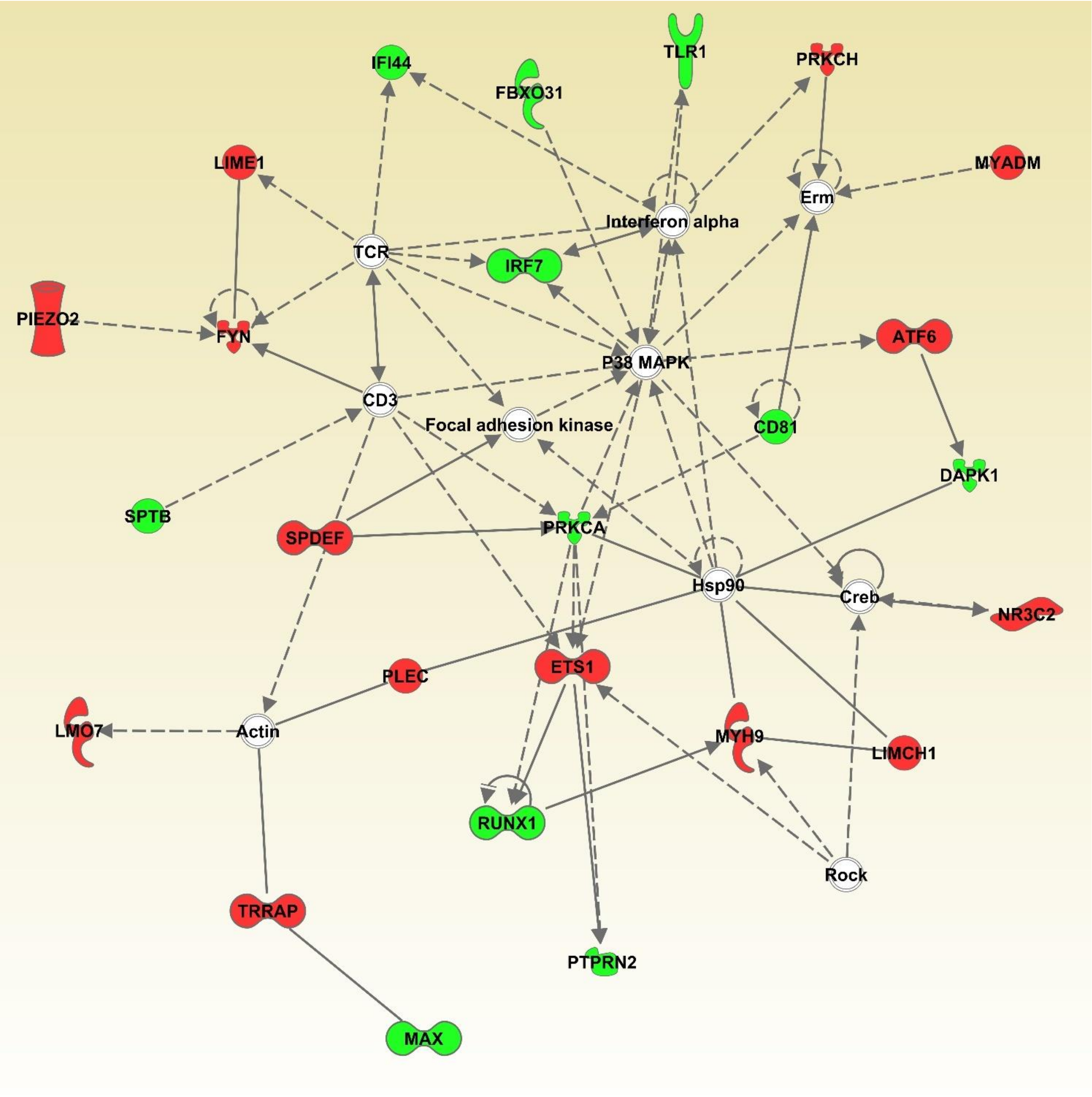

Supplementary Figure 3: SLEDAI score distribution in African-American and European-American lupus patients at initial sample collection.

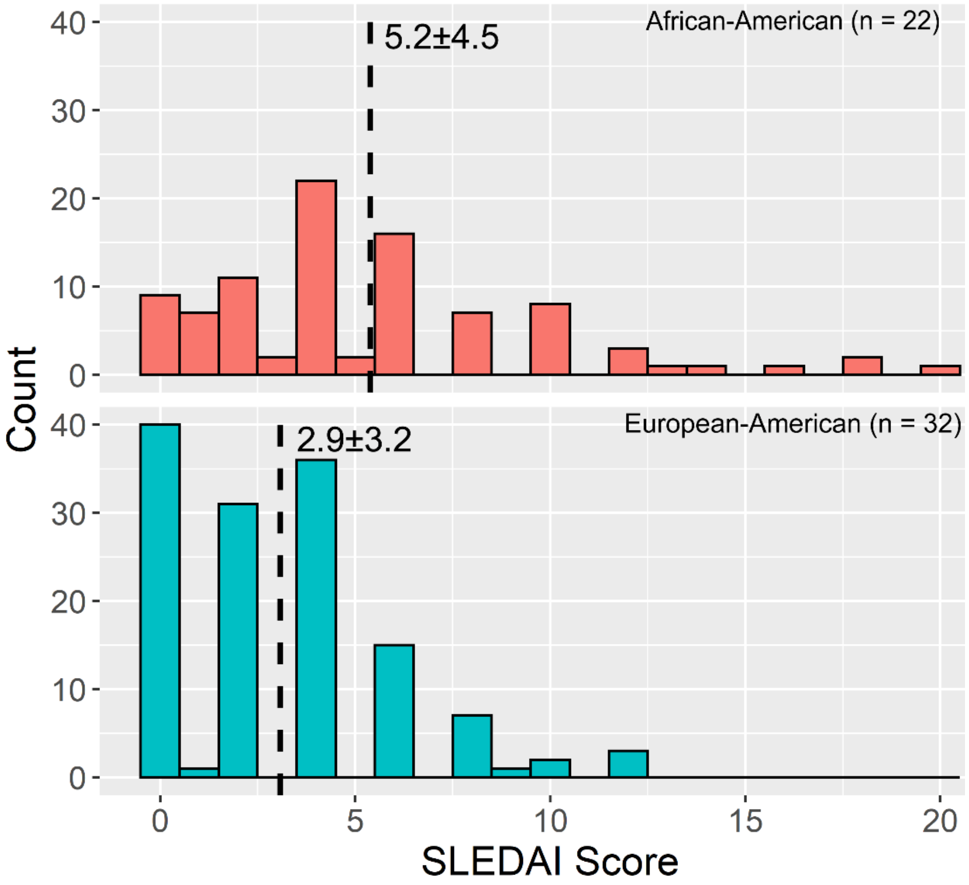

Supplementary Figure 4. Dot plot showing correlation of mean DNA methylation for 71 neutrophil-specific probes from purified cell populations in Salas *et al.*(1) and mean DNA methylation of the same probes in isolated granulocytes in our lupus cohort (n = 229). Pearson's product-moment correlation test had a *r* = 0.996 and P-value < 2.2E-16.

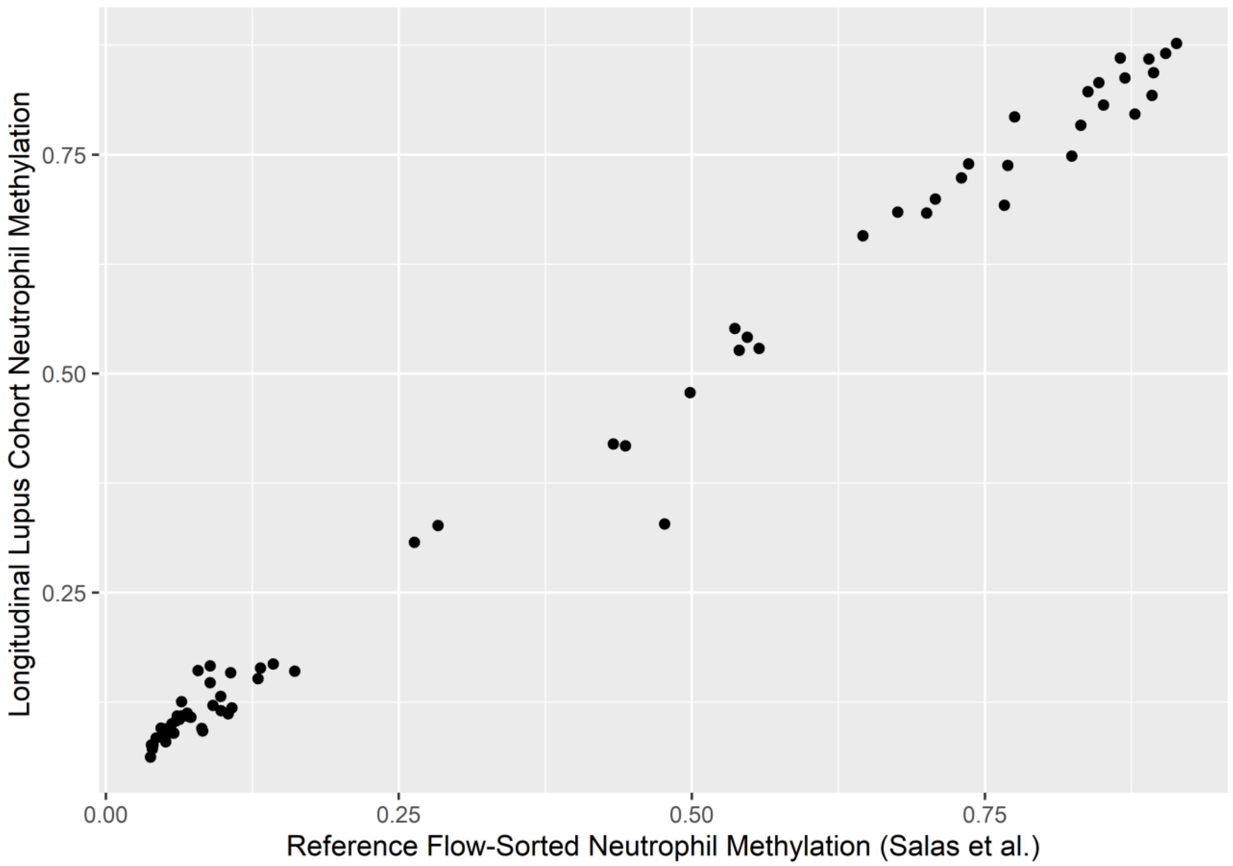

Supplementary Figure 5: Dot plot of top two genotype principal components. Grey squares represent European-American lupus patients and black dots represent African-American lupus patients.

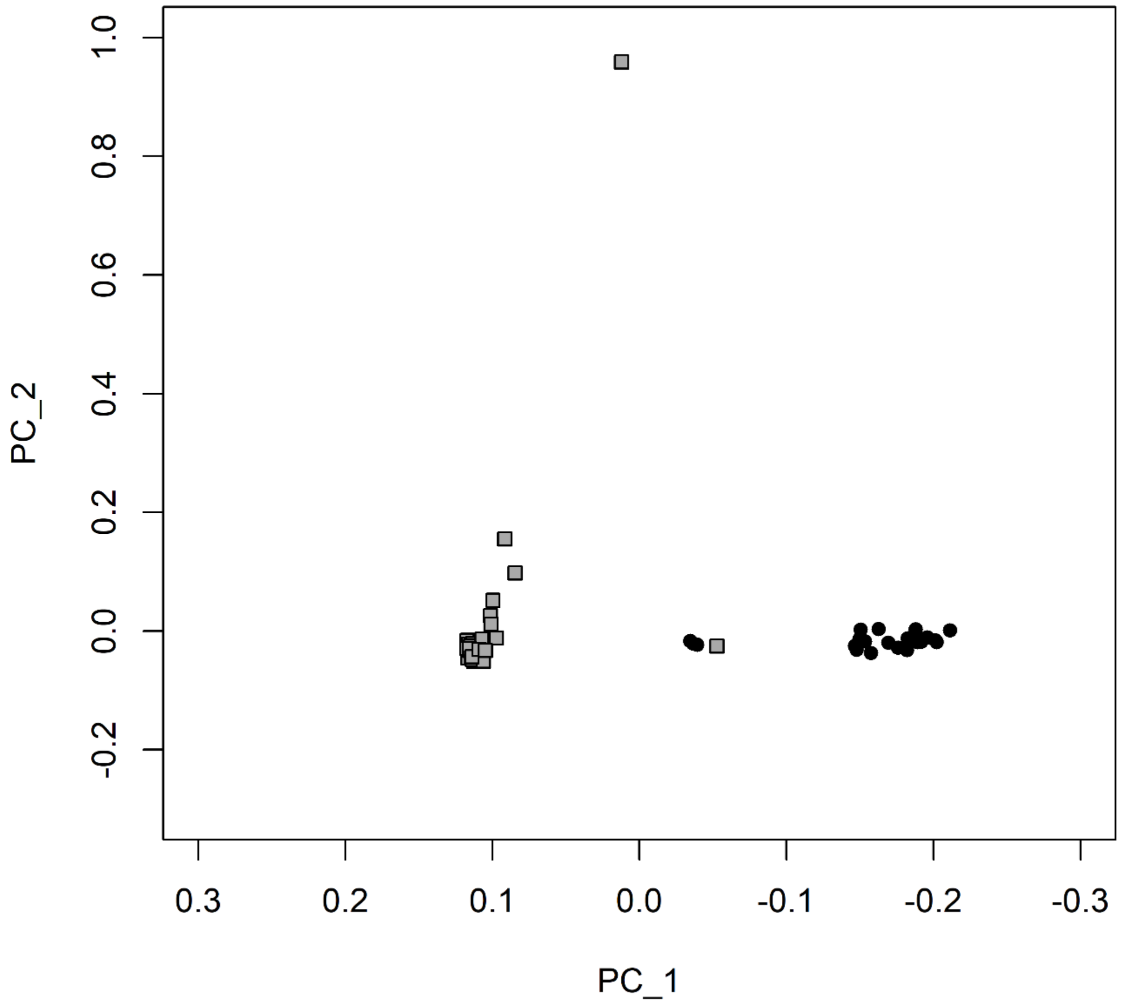

Reference

1. Salas LA, Koestler DC, Butler RA, Hansen HM, Wiencke JK, Kelsey KT, et al. An optimized library for reference-based deconvolution of whole-blood biospecimens assayed using the Illumina HumanMethylationEPIC BeadArray. *Genome Biol.* 2018;19(1):64.
